# EValuating the Effect of periopeRaTIve empaGliflOzin on cardiac surgery associated acute kidney injury: rationale and design of the VERTIGO study

**DOI:** 10.1093/ckj/sfae229

**Published:** 2024-07-22

**Authors:** Armando Coca, Elena Bustamante-Munguira, Verónica Fidalgo, Manuel Fernández, Cristina Abad, Marta Franco, Ángel González-Pinto, Daniel Pereda, Sergio Cánovas, Juan Bustamante-Munguira

**Affiliations:** Department of Nephrology, Hospital Clínico Universitario, Valladolid, Spain; Department of Medicine, Dermatology, and Toxicology, Facultad de Medicina, Universidad de Valladolid, Valladolid, Spain; Department of Medicine, Dermatology, and Toxicology, Facultad de Medicina, Universidad de Valladolid, Valladolid, Spain; Department of Intensive Care Medicine, Hospital Clínico Universitario, Valladolid, Spain; Department of Nephrology, Hospital Virgen de la Concha, Zamora, Spain; Department of Cardiovascular Surgery, Hospital Clínico Universitario Virgen de la Arrixaca, Murcia, Spain; Department of Immunology, Hospital Clínico Universitario, Valladolid, Spain; Department of Intensive Care Medicine, Hospital Clínico Universitario, Valladolid, Spain; Department of Cardiovascular Surgery, Hospital General Universitario Gregorio Marañón, Madrid, Spain; Department of Cardiovascular Surgery, Hospital Clinic de Barcelona, Barcelona, Spain; Department of Cardiovascular Surgery, Hospital Clínico Universitario Virgen de la Arrixaca, Murcia, Spain; Department of Cardiovascular Surgery, Hospital Clínico Universitario, Valladolid, Spain; Department of Surgery, Facultad de Medicina, Universidad de Valladolid, Valladolid, Spain

**Keywords:** acute kidney injury, cardiac surgery, ischemia, ischemia–reperfusion injury, survival analysis

## Abstract

**Background:**

Cardiac surgery-associated acute kidney injury (CSA-AKI) is a serious complication in patients undergoing cardiac surgery with extracorporeal circulation (ECC) that increases postoperative complications and mortality. CSA-AKI develops due to a combination of patient- and surgery-related risk factors that enhance renal ischemia–reperfusion injury. Sodium-glucose cotransporter 2 inhibitors (SGLT2i) such as empagliflozin reduce renal glucose reabsorption, improving tubulo-glomerular feedback, reducing inflammation and decreasing intraglomerular pressure. Preclinical studies have observed that SGLT2i may provide significant protection against renal ischemia–reperfusion injury due to their effects on inadequate mitochondrial function, reactive oxygen species activity or renal peritubular capillary congestion, all hallmarks of CSA-AKI. The VERTIGO (EValuating the Effect of periopeRaTIve empaGliflOzin) trial is a Phase 3, investigator-initiated, randomized, double-blind, placebo-controlled, multicenter study that aims to explore whether empagliflozin can reduce the incidence of adverse renal outcomes in cardiac surgery patients.

**Methods:**

The VERTIGO study (EudraCT: 2021-004938-11) will enroll 608 patients that require elective cardiac surgery with ECC. Patients will be randomly assigned in a 1:1 ratio to receive either empagliflozin 10 mg orally daily or placebo. Study treatment will start 5 days before surgery and will continue during the first 7 days postoperatively. All participants will receive standard care according to local practice guidelines. The primary endpoint of the study will be the proportion of patients that develop major adverse kidney events during the first 90 days after surgery, defined as ≥25% renal function decline, renal replacement therapy initiation or death. Secondary, tertiary and safety endpoints will include rates of AKI during index hospitalization, postoperative complications and observed adverse events.

**Conclusions:**

The VERTIGO trial will describe the efficacy and safety of empagliflozin in preventing CSA-AKI. Patient recruitment is expected to start in May 2024.

## Graphical Abstract

**Graphical Abstract:**
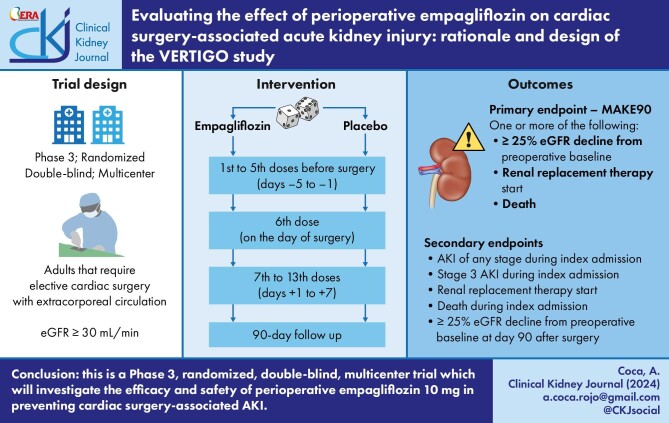

KEY LEARNING POINTS **What was known:**

- Cardiac surgery-associated acute kidney injury (CSA-AKI) is a life-threatening complication that affects almost half of patients undergoing cardiac surgery with extracorporeal circulation and is associated with higher incidence of postoperative complications and mortality.
- Although several preventive strategies have been tested to reduce the high rates of CSA-AKI, none has achieved convincing enough results to integrate these therapies in daily clinical practice.

**This study adds:**

- Sodium-glucose cotransporter 2 inhibitors are a class of oral antidiabetic drugs that have demonstrated significant nephroprotective effects, slowing the progression of chronic kidney disease, as well as reducing proteinuria.
- The VERTIGO (EValuating the Effect of periopeRaTIve empaGliflOzin) trial aims to describe the effect of perioperative empagliflozin to reduce the incidence of CSA-AKI in patients that require elective cardiac surgery.

**Potential impact:**

- The trial will provide evidence about the efficacy and safety of empagliflozin in reducing the incidence and severity of CSA-AKI.
- If results are favorable, the planned intervention could be widely applied as a preventive therapeutic strategy in this clinical setting.

## INTRODUCTION

Cardiac surgery-associated acute kidney injury (CSA-AKI) is a serious complication that affects up to 40% of patients undergoing cardiac surgery with extracorporeal circulation (ECC). This complication is associated with higher incidence of postoperative complications and mortality, longer in-hospital stay and increased healthcare costs [1].

The combination of risk factors such as age, patient comorbidities such as hypertension, diabetes or peripheral artery disease, together with other determinants associated with surgery itself, such as time on ECC, aortic cross-clamp time or high-dose inotrope administration, explain the high incidence of CSA-AKI [2]. The joint action of these risk factors induces systemic and renal tissue ischemia, increased oxidative stress and inflammation [3].

Kidneys are especially vulnerable to ischemia, which occurs in two phases: during the initial phase, ischemia triggers nutrient and oxygen supply reduction, causing kidney cell injury and death [4]. An intense inflammatory response develops during the second, reperfusion phase, which intensifies tissue damage and leads to acute renal failure [5].

Over the last few years, several preventive strategies have been tested aiming at reducing the high rates of CSA-AKI and its consequences, including the use of albumin, erythropoietin or remote ischemic preconditioning, without achieving conclusive enough results to allow the integration of these therapies in daily clinical practice [6–8].

Sodium-glucose cotransporter 2 inhibitors (SGLT2i) are a class of oral antidiabetic drugs that inhibit renal glucose reabsorption in the proximal tubule of the nephron, increasing renal glucose excretion, improving tubulo-glomerular feedback by inducing efferent arteriole vasodilation and decreasing intraglomerular pressure. The consequence of these changes is a significant short- and long-term nephroprotective effect [9, 10].

SGLT2i induce a fasting-like state that facilitates fatty acid β-oxidation, body adiposity reduction and ketone formation [11]. Glycosuria resulting from SGLT2i administration reduces plasma glucose and insulin levels, creating an energy-deficient metabolic state that stimulates the synthesis of ketones such as β-hydroxybutyrate that can be used as an alternative energy source. This molecule has significant antioxidant and anti-inflammatory effects due to its capacity to activate the hydroxycarboxylic acid receptor 2, stimulate anti-inflammatory macrophage subpopulations and the formation of prostaglandin E2, or modulate the activity of antioxidant enzymes such as catalase or manganese superoxide dismutase [9, 12–14]. β-Hydroxybutyrate is also capable of protecting mitochondrial function and inhibiting hydroxyl radical activity that boosts oxidative stress–associated damage [15, 16]. Depleted levels of prostaglandin E2 or superoxide dismutase 1 concentrations in kidney tissue, as well as reduced mitochondrial abundance, have been associated with more severe ischemic AKI [17–19].

In parallel to their effect on ketone formation, SGLT2i have additional anti-inflammatory properties that generate synergistic effects, such as the inhibition of the NOD-like receptor Pyrin domain containing protein 3 (NLRP3) inflammasome, the modulation of metabolites of the tricarboxylic acid cycle or the reduction of reactive oxygen species activity [20, 21]. Persistent overexpression of NLRP3 has been associated with the severity of ischemia–reperfusion-induced AKI [22]. In animal models of renal ischemia, SGLT2i stimulated the expression of hypoxia-inducible factor 1 (HIF-1), reducing the Bax/Bcl2 ratio in renal tissue, improving renal function and reducing apoptosis [23]. It has also been observed that SGLT2i prevented ischemia–reperfusion-associated renal peritubular capillary congestion and hemorrhage [24].

Results of the recent EMPA-KIDNEY (The study of heart and kidney protection with empagliflozin) and DAPA-CKD (A study to evaluate the effect of dapagliflozin on renal outcomes and cardiovascular mortality in patients with chronic kidney disease) trials have demonstrated the ability of SGLT2i to reduce proteinuria in chronic kidney disease, considerably slowing its progression [25, 26]. The fundamentals for such effect, including downregulation of proinflammatory and profibrotic pathways in the tubule or decreased energy expenditure via Na+/K+ ATPase and Na+/H+ exchanger 3, could perfectly well apply to ischemic AKI [27].

SGLT2i have shown great preclinical potential against ischemia–reperfusion injury in multiple organs—including the kidney—and with a wide administration window, from 7 days to 2 h before ischemia–reperfusion injury. This effect seems to be mediated through several mechanisms, such as increased renal levels of glycogen synthase kinase 3β, restoration of urinary levels of microRNA-26a, or a downregulation in ischemic tissue of the expression of proinflammatory markers such as interleukin-1, interleukin-6 or tumor necrosis factor-α [28–30].

There are no published data regarding key points of the study, such as the minimum effective dose of empagliflozin as a preventive therapy for CSA-AKI or the most appropriate timing for therapy initiation. Furthermore, SGLT2i have been considered as “sick days drugs,” with some guidelines recommending against their routine use during hospital stays [31]. Nevertheless, results of empagliflozin administration in patients admitted in intensive care/cardiac care units have already been reported, with an acceptable safety profile [32, 33]. The results of our study will provide key data regarding the efficacy and safety of empagliflozin in this setting. A dose of empagliflozin 10 mg has been chosen, since it has shown both long-term nephro- and cardio-protective efficacy, with a low incidence of adverse effects [25, 34].

The aim of this trial is to analyze the effect of the perioperative administration of empagliflozin 10 mg in patients who will undergo elective cardiac surgery with ECC on kidney function, inflammation, oxidative stress, postoperative complications and mortality. To do this, we have designed a Phase 3, investigator-initiated, randomized, double-blind, placebo-controlled, multicenter study.

## MATERIALS AND METHODS

### Study objective

The primary aim of the VERTIGO (EValuating the Effect of periopeRaTIve empaGliflOzin) trial is to evaluate whether perioperative empagliflozin reduces the composite endpoint of worsening of renal function, defined as a decline in estimated glomerular filtration rate (eGFR) of ≥25%, renal replacement therapy initiation or death during the first 90 days following surgery, compared with placebo, in patients requiring elective cardiac surgery with ECC. Furthermore, the trial will evaluate the effects of empagliflozin, compared with placebo, on postoperative AKI severity, postoperative complications, length of intensive care unit and in-hospital stay, as well as need for hospital readmission for cardiovascular disease, patient-reported quality of life and safety profile of empagliflozin in this setting. This study will be conducted in accordance with the declaration of Helsinki and the International Council for Harmonisation Guidelines on Good Clinical Practice, and has been designed following the recommendations of the COMET Handbook and the SPIRIT 2013 Statement [35, 36]. The first patient will be enrolled in May 2024 and study completion is expected in approximately December 2025. The trial is registered with https://eudract.ema.europa.eu (EudraCT: 2021-004938-11).

### Study design

**Figure 1:**
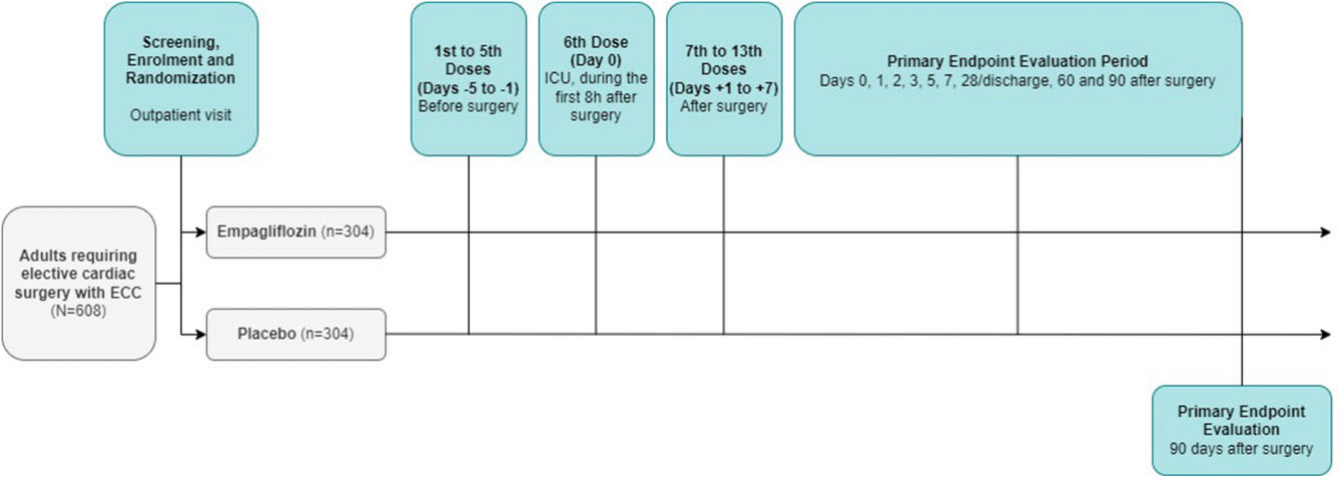
VERTIGO study design. ICU, intensive care unit.

VERTIGO is a Phase 3, investigator-initiated, randomized, double-blind, placebo-controlled, multicenter study that will recruit 608 patients at four third-level, academic hospitals. Figure 1 shows the overall study design.

### Trial participants

Trial participants will be adults that require elective cardiac surgery with ECC with a baseline eGFR >30 mL/min/1.73 m^2^. Added inclusion and exclusion criteria are indexed in Table 1.

**Table 1:** Inclusion and exclusion criteria.


Inclusion criteria
- ≥18 years of age
- Requiring elective cardiac surgery with ECC
- Able to understand the aim of the study and to provide written informed consent
Exclusion criteria
- Emergent surgery
- Endocarditis
- Patients awaiting heart transplantation
- Planned use of intra-aortic balloon pump
- Type 1 diabetes
- Chronic kidney disease with eGFR <30 mL/min/1.73 m^2^
- Severe liver dysfunction (ALT or AST >3 times above the upper limit of normality)
- Active cancer
- Active treatment with any SGLT2i
- Known allergy to empagliflozin, any other SGLT2i or any of its excipients
- Solid organ transplant recipients
- Pregnant or lactating women
- Patients who are participating in another clinical trial

ALT, alanine transaminase; AST, aspartate transaminase.

### Enrolment

Potentially eligible patients will be invited for screening. Those who meet all inclusion criteria, and no exclusion criteria will be randomized within 3 days after the screening visit.

### Randomization, stratification and blinding

Patients meeting all eligibility criteria will be randomized in a 1:1 ratio to receive either empagliflozin 10 mg orally daily or matching placebo in a double-blind manner. These patients will constitute the primary intention-to-treat population for evaluating the efficacy and safety of the planned intervention. Randomization will be performed centrally employing an interactive web system with a computer-generated randomization schedule and will be stratified according to study site and diabetic status (yes/no) to ensure a similar proportion of patients with and without type 2 diabetes in both treatment groups. Block randomization with random block sizes will be employed to minimize selection bias. Planned enrolment is 608 patients from four sites.

All patients and study personnel, except for the independent data-monitoring committee, will be kept blinded to treatment allocation. Empagliflozin and placebo will be packaged in an indistinguishable manner, with identical appearance and labelling. Study medication will be administered in the morning, except for the sixth dose (first postoperative dose), at approximately the same time of the day throughout the study.

### Schedule of assessments

The first study visit will be performed immediately after study participants provide written informed consent, enrolment and randomization. Anthropometric information, baseline treatment and laboratory data will be recorded. Elective cardiac surgery will be programmed within 30 days of the first visit. The research team will provide study medication to each participant. Doses 1–5 of study medication will be administered daily on the last 5 days before surgery (Days –5 to –1). The sixth dose will be administered within the first 8 h after surgery (Day 0). Doses 7–13 will be administered daily during the first 7 days after surgery (Fig. 1). Previous research has not adequately defined the best timing for SGLT2i initiation to prevent ischemia–reperfusion injury with protocols starting SGLT2i administration both before and after the onset of injury [21, 23, 24]. However, empagliflozin has been shown to reach the peak of its glycosuric effect within the first 24 h after its administration, with further stabilization over the next 72 h. Moreover, renal protective effects of SGLT2i seem to appear early after treatment initiation [23, 37]. We chose to start study medication 5 days before surgery to ensure a stable and sustained effect of empagliflozin on the kidney.

**Figure 2:**
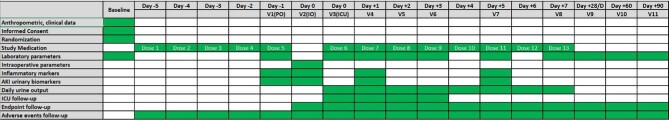
Study visit schedule. D, discharge; ICU, intensive care unit; IO, intraoperative; PO, preoperative.

The visit schedule is summarized in Fig. 2. Vital signs, blood and urine samples, as well as information about study endpoints, adverse events and other outcomes of interest, will be recorded in each visit.

Discontinuation of the study drug will be required for patients who develop any serious adverse event that could reasonably be related to study medication, at the discretion of the treating physician. Patients who prematurely discontinue the study drug but do not withdraw consent will continue follow-up visits as scheduled if possible.

### Outcome definitions and background medication

The primary endpoint of the study will be the time to the first occurrence of any major adverse kidney event during the first 90 days after surgery (MAKE90). The components of MAKE90 will be a ≥25% eGFR decline from preoperative baseline, the start of renal replacement therapy or patient death. Secondary, tertiary and safety endpoints are listed in Table 2. Treating physicians will be responsible for adjudicating possible postoperative complications.

**Table 2:** Primary, secondary, tertiary and safety endpoint of the VERTIGO trial.


Primary endpoint
Proportion of patients that present MAKE90, defined as one or more of the following:
- ≥25% eGFR decline from preoperative baseline
- Start of renal replacement therapy
- Patient death
Secondary endpoints
- Proportion of participants who developed any stage of AKI defined according to KDIGO guidelines during index admission
- Proportion of patients who suffered a Stage 3 AKI according to KDIGO guidelines during index admission
- Proportion of patients who required renal replacement therapy during index admission
- Proportion of patients who died during index admission
- Proportion of patients that maintain ≥25% eGFR decline from preoperative baseline 90 days after surgery
Tertiary endpoints
- Difference in total time (h) on mechanical ventilation
- Difference in mean daily dose of inotropes used
- Difference in plasma levels of interleukin-6 and C-reactive protein
- Difference in urinary levels of interleukin-18 and protein/creatinine ratio
- Difference in serum interleukin-1β and interleukin-18 concentration
- Time to first occurrence of postoperative complications, defined as low cardiac output, reoperation for bleeding, delirium, seizures, surgical site infection, sepsis, arrhythmias, cardiac arrest, pneumonia, liver dysfunction or need for blood transfusion
- Difference in total time (days) spent in the ICU
- Difference in total time (days) of index hospital stay
- Difference in rates of hospital readmission due to cardiovascular disease
- Difference in frailty evaluation, represented by the results of the Clinical Frailty Scale
- Patient reported quality of life EuroQoL-5D
Safety endpoints
- Adverse events
- Serious adverse events (i.e. adverse event that results in death, is considered life-threatening, requires or extends an hospitalization episode, causes disability or is judged as such according to the clinical criteria of the research team)
- Discontinuation of study medication due to adverse events

ICU, intensive care unit.

Adverse event data will be recorded. Serious adverse events and adverse events that lead to premature study medication discontinuation or interruption will be immediately reported. Background medication will be left to the discretion of treating physicians, in accordance with local clinical practice guidelines. Postoperative management will be performed according to each center's protocol to ensure a real-world approach.

### Sample size calculation and statistical analyses

Previous studies have observed an incidence of MAKE90 of 25% among patients that required elective cardiac surgery [38, 39]. Assuming the prior proportion of MAKE90 to be 25% for the placebo group, a sample size of 546 patients (273 patients per treatment group) will provide a 90% (β = 0.10) power to detect a statistically significant difference of 10% in the proportion of patients with MAKE90 under a one-sided significance level of 5% (α = 0.05), i.e. to detect a proportion of 15% for the empagliflozin group. Finally, by taking in account a potential dropout rate of 10% we determined a sample size of 608 patients (304 patients per treatment group).

Our trial will apply adaptive Bayesian methodology to estimate the proportions of patients with the primary endpoint in both groups. For such a purpose, a conjugate beta-binomial model will be used, allowing for continuous updates of posterior distributions as new samples are obtained. This means that, as additional data are collected, proportion estimates will be dynamically adjusted, providing a real-time view of treatment effectiveness in disease prevention.

Two interim analyses will be carried out by the independent data monitoring committee. These analyses will be carried out once 30% and 60% of the planned study sample have been recruited. The objective of the first analysis will be to assess the futility of the study intervention, using conditional power analysis. The aim of the second analysis will be to evaluate the need to adjust the study sample size. Both interim analyses will be performed by an external statistician to ensure that no loss of blinding occurs.

Primary endpoint analysis will be based on the intention-to-treat population, defined as all validly randomized patients. In the analysis of the primary endpoint, study treatments will be compared using a Cox proportional hazards regression model with a factor for the treatment group. Hazard ratio, 95% confidence interval and *P*-value will be reported. Kaplan–Meier estimates of the cumulative incidence to the occurrence of MAKE90 will be calculated. Secondary endpoints will be tested in a similar manner. If superiority is achieved for the primary endpoint, the secondary endpoints will be tested following a hierarchical order, as follows. (i) Any stage of AKI defined according to Kidney Disease: Improving Global Outcomes (KDIGO) guidelines during index admission. (ii) Stage 3 AKI according to KDIGO guidelines during index admission. (iii) Time to renal replacement therapy initiation during index admission. (iv) Time to death from any cause. Statistical significance will be required before testing the next hypothesis in the hierarchy. The effects of the planned intervention on primary and secondary outcomes will also be assessed in subanalyses stratified by sex, baseline eGFR, diabetic status and type of planned surgery (valvular/coronary/valvular and coronary).

## DISCUSSION

VERTIGO is designed as a Phase 3, investigator-initiated, randomized, double-blind, placebo-controlled, multicenter study that will examine whether empagliflozin added to standard care for patients that undergo elective cardiac surgery reduces the incidence of MAKE up to 90 days after surgery.

CSA-AKI is a common complication that occurs in approximately 20%–40% of patients that require cardiac surgery [40]. Its incidence is expected to increase in the coming years due to progressive changes in the profile of these patients, who are older and suffer greater comorbidities. Multiple perioperative strategies have been implemented to reduce the incidence of kidney-related adverse events associated with cardiac surgery. These include pharmacological, technical and management-related interventions, performed pre-, intra- and/or postoperatively [40]. Published trials and meta-analyses are characterized by a marked lack of homogeneity in renal outcomes definition, timing of planned intervention as well as length of follow-up.

Certain interventions, such as perioperative sodium bicarbonate, n-acetylcysteine, dexmedetomidine or remote ischemic preconditioning have proved ineffective [8, 41, 42]. In addition, several randomized controlled trials have offered conflicting results regarding actual efficacy of specific interventions, like the administration of erythropoietin or levosimendan [7, 43, 44]. Additional controlled trials that were studying potential treatment alternatives, such as hemoperfusion with CytoSorb during ECC (NCT3384875) or teprasiran (NCT03510897), were terminated early due to either undisclosed reasons or to results not meeting efficacy outcomes at the prespecified time point.

As a result, no preventive or therapeutic strategy for CSA-AKI, outside of hemodynamic, respiratory and nutritional support, has managed to effectively translate so far into daily clinical practice, while morbidity and mortality remain significantly increased not only in the acute postoperative period but also in the ensuing years after surgery, generating index hospitalization-associated costs of approximately US ${\$}$1.01 billion annually in the USA [45, 46]. In this study we seek to test the potential of perioperative empagliflozin in preventing CSA-AKI and MAKE90.

Several studies have observed incidence rates of MAKE90 in cardiac surgery patients of approximately 25% [38, 39]. A sample size of 608 patients will provide power of 90% to demonstrate a 10% reduction in the incidence of MAKE90 with empagliflozin versus placebo. This proportion has been chosen as it has been considered potentially achievable after reviewing the preclinical data and results of previously conducted trials on the subject, in addition to considering it as a clinically significant reduction [28, 44, 45]. Due to the existing limitations in relation to previous data, two interim analyses have been planned to evaluate futility and the need for a sample size re-estimation, applying adaptive Bayesian methodology. Results of the interim analyses will be reviewed by an independent data-monitoring committee, who will provide a recommendation to stop the trial early due to futility or to maintain or increase the sample size. All research team members, as well as participants, will remain blinded until study completion.

The primary endpoint of the study will be the incidence of MAKE during the first 90 days after surgery. This endpoint has been considered as a clinically meaningful tool that may help to increase our capacity to understand AKI in a wide variety of clinical settings, as well as providing a composite to allow comparison of potential interventions [47]. MAKE90 combines worsened renal function, defined as a decline in eGFR of ≥25%, new dialysis or death, thus including new-onset or progressive renal dysfunction of variable intensity, as well as death as the definitive major morbid outcome. A renal composite such as MAKE90 allows assessment of renal disability-free survival, securing a higher proportion of patients that suffer poor renal outcomes, in addition to eliminating potential competing risks [47].

Secondary endpoints will provide additional information on the effect of empagliflozin on the incidence of AKI immediately after cardiac surgery. The use of KDIGO consensus criteria for AKI, a classification that employs serum creatinine levels as well as urine output to classify AKI severity [48], will allow for easier comparison of efficacy across different therapeutic strategies. The description of the effect of the planned intervention on kidney function will be completed with urinary biomarkers of AKI such as interleukin-18 or DKK3/albumin ratio. The results of secondary endpoints, in combination with the primary endpoint of the study, will provide an extensive exploration of the efficacy of empagliflozin in preventing CSA-AKI in the study sample using clinically meaningful outcomes.

Tertiary endpoints, such as time on mechanical ventilation after surgery, average inotrope dose, biomarkers of inflammation and AKI, frailty assessment, incidence of postoperative complications, as well as total length of intensive care unit and hospital stay, or need for hospital readmission for cardiovascular disease, will allow us to analyze the short- and medium-term postoperative recovery of patients.

The study will also provide information on the safety of perioperative empagliflozin in patients that require elective cardiac surgery. The safety profile of SGLT2i, and specifically empagliflozin, has been extensively described in recent years, both in patients with diabetes as well as in non-diabetic chronic kidney disease [25, 49, 50]. Empagliflozin is generally well tolerated, and specific potential complications, such as symptomatic dehydration, hypoglycemia or urinary tract infections, will be carefully monitored and reported throughout the trial.

Although multiple interventions have been tested aiming to reduce the incidence and severity of CSA-AKI, no treatment strategy has been successfully translated into daily clinical practice. Therefore, our study will be placebo controlled. All participants in the study will receive standard treatment as per treating physicians and local practice guidelines. Randomization will be stratified by center and diabetic status. To minimize the risk of selection bias, block randomization with random block sizes will be employed.

In summary, this trial will provide evidence about the efficacy and safety of empagliflozin in reducing the incidence and severity of CSA-AKI in patients that require elective cardiac surgery. To accomplish this task, we will use a Phase 3, investigator-initiated, randomized, double-blind, placebo-controlled, multicenter design, using standard care in both study groups and analyzing clinically relevant primary, secondary and tertiary endpoints. Interim analysis will be conducted with the purpose of assessing early futility and the need for sample size adjustment. Patient recruitment is expected to start in May 2024.

## Data Availability

No new data were generated or analysed in support of this research.

## References

[1] O'Neal JB, Shaw AD, Billings FT. Acute kidney injury following cardiac surgery: current understanding and future directions. Crit Care 2016;20:187. 10.1186/s13054-016-1352-z 27373799 PMC4931708

[2] Lopez-Delgado JC, Esteve F, Torrado H et al. Influence of acute kidney injury on short- and long-term outcomes in patients undergoing cardiac surgery: risk factors and prognostic value of a modified RIFLE classification. Crit Care 2013;17:R293. 10.1186/cc13159 24330769 PMC4056889

[3] Gomez H, Ince C, De Backer D et al. A unified theory of sepsis-induced acute kidney injury: inflammation, microcirculatory dysfunction, bioenergetics, and the tubular cell adaptation to injury. Shock 2014;41:3–11. 10.1097/SHK.0000000000000052 PMC3918942 24346647

[4] Schrier RW, Wang W, Poole B et al. Acute renal failure: definitions, diagnosis, pathogenesis, and therapy. J Clin Invest 2004;114:5–14. 10.1172/JCI22353 15232604 PMC437979

[5] Eltzschig HK, Eckle T. Ischemia and reperfusion—from mechanism to translation. Nat Med 2011;17:1391–401. 10.1038/nm.2507 22064429 PMC3886192

[6] Lee EH, Kim WJ, KIM JY et al. Effect of exogenous albumin on the incidence of postoperative acute kidney injury in patients undergoing off-pump coronary artery bypass surgery with a preoperative albumin level of less than 4.0 g/dl. Anesthesiology 2016;124:1001–11. 10.1097/ALN.0000000000001051 26891150

[7] Dardashti A, Ederoth P, Algotsson L et al. Erythropoietin and protection of renal function in cardiac surgery (the EPRICS Trial). Anesthesiology 2014;121:582–90. 10.1097/ALN.0000000000000321 25225746

[8] Hausenloy DJ, Candilio L, Evans R et al. Remote ischemic preconditioning and outcomes of cardiac surgery. N Engl J Med 2015;373:1408–17. 10.1056/NEJMoa1413534 26436207

[9] Tsimihodimos T, Filippatos TD, Elisaf MS. SGLT2 inhibitors and the kidney: effects and mechanisms. Diabetes Metab Syndr 2018;12:1117–23. 10.1016/j.dsx.2018.06.003 29909004

[10] Menne J, Dumann W, Haller H et al. Acute kidney injury and adverse renal events in patients receiving SGLT2-inhibitors: a systematic review and meta-analysis. PLoS Med 2019;16:e1002983. 10.1371/journal.pmed.1002983 31815931 PMC6901179

[11] Osataphan S, Macchi C, Singhal G et al. SGLT2 inhibition reprograms systemic metabolism via FGF21-dependent and -independent mechanisms. JCI Insight 2019;4:e123130. 10.1172/jci.insight.123130 30843877 PMC6483601

[12] Rahman M, Muhammad S, Khan MA et al. The beta-hydroxybutyrate receptor HCA2 activates a neuroprotective subset of macrophages. Nat Commun 2014;5:3944. 10.1038/ncomms4944 24845831

[13] Tran MT, Zsengeller ZK, Berg AH. PGC1α drives NAD biosynthesis linking oxidative metabolism to renal protection. Nature 2016;531:528–32. 10.1038/nature17184 26982719 PMC4909121

[14] Li L, Kang H, Zhang Q et al. FoxO3 activation in hypoxic tubules prevents chronic kidney disease. J Clin Invest 2019;129:2374–89. 10.1172/JCI122256 30912765 PMC6541430

[15] Zhang M, Dong W, Li Z et al. Effect of forkhead box O1 in renal tubular epithelial cells on endotoxin-induced acute kidney injury. Am J Physiol Renal Physiol 2021;320:F262–72. 10.1152/ajprenal.00289.2020 33356954

[16] Tumlin J, Stacul F, Adam A et al. Pathophysiology of contrast-induced nephropathy. Am J Cardiol 2006;98:14K–20K. 10.1016/j.amjcard.2006.01.020 16949376

[17] Kim HJ, Kim SH, Kim M et al. Inhibition of 15-PGDH prevents ischemic renal injury by the PGE2/EP4 signaling pathway mediating vasodilation, increased renal blood flow, and increased adenosine/A2A receptors. Am J Physiol Renal Physiol 2020;319:F1054–66. 10.1152/ajprenal.00103.2020 33135478 PMC7792690

[18] Holthoff JH, Harville Y, Herzog C et al. SOD1 is a novel prognostic biomarker of acute kidney injury following cardiothoracic surgery. BMC Nephrol 2023;24:299. 10.1186/s12882-023-03350-8 37821813 PMC10568797

[19] Jotwani V, Thiessen-Philbrook H, Arking DE et al. Association of blood mitochondrial DNA copy number with risk of acute kidney injury after cardiac surgery. Am J Kidney Dis 2024;S0272-6386(24)00719-4. 10.1053/j.ajkd.2024.03.013 PMC12416630 38640995

[20] Kim SR, Lee SG, Kim SH et al. SGLT2 inhibition modulates NLRP3 inflammasome activity via ketones and insulin in diabetes with cardiovascular disease. Nat Commun 2020;11:2127. 10.1038/s41467-020-15983-6 32358544 PMC7195385

[21] Ke Q, Shi C, Lv Y et al. SGLT2 inhibitor counteracts NLRP3 inflammasome via tubular metabolite itaconate in fibrosis kidney. FASEB J 2022;36:e22078. 10.1096/fj.202100909RR 34918381

[22] Zheng Z, Xu K, Li C et al. NLRP3 associated with chronic kidney disease progression after ischemia/reperfusion-induced acute kidney injury. Cell Death Discov 2021;7:324. 10.1038/s41420-021-00719-2 34716316 PMC8556399

[23] Chang YK, Choi H, Jeong JY et al. Dapagliflozin, SGLT2 inhibitor, attenuates renal ischemia-reperfusion injury. PLoS One 2016;11:e0158810. 10.1371/journal.pone.0158810 27391020 PMC4938401

[24] Zhang Y, Nakano D, Guan Y et al. A sodium-glucose transporter 2 inhibitor attenuates renal capillary injury and fibrosis by a vascular endothelial growth factor–dependent pathway after renal injury in mice. Kidney Int 2018;94:524–35. 10.1016/j.kint.2018.05.002 30045814

[25] Herrington WG, Staplin N, Wanner C et al. Empagliflozin in patients with chronic kidney disease. N Engl J Med 2023;388:117–27. 10.1056/NEJMoa2204233 36331190 PMC7614055

[26] Heerspink HJL, Stefánsson BV, Correa-Rotter R et al. Dapagliflozin in patients with chronic kidney disease. N Engl J Med 2020;383:1436–46. 10.1056/NEJMoa2024816 32970396

[27] Skrabic R, Kumric M, Vrdoljak J et al. SGLT2 inhibitors in chronic kidney disease: from mechanisms to clinical practice. Biomedicines 2022;10:2458. 10.3390/biomedicines10102458 36289720 PMC9598622

[28] Wang Q, Ju F, Liu T et al. Empagliflozin protects against renal ischemia/reperfusion injury in mice. Sci Rep 2022;12:19323. 10.1038/s41598-022-24103-x 36369319 PMC9652474

[29] Chu C, Delic D, Alber J et al. Head-to-head comparison of two SGLT-2 inhibitors on AKI outcomes in a rat ischemia-reperfusion model. Biomed Pharmacother 2022;153:113357. 10.1016/j.biopha.2022.113357 35792391

[30] Gokbulut P, Kuskonmaz SM, Koc G et al. Evaluation of the effects of empagliflozin on acute lung injury in rat intestinal ischemia-reperfusion model. J Endocrinol Invest 2023;46:1017–26. 10.1007/s40618-022-01978-1 36495440

[31] American Diabetes Association . 15. Diabetes care in the hospital: standards of medical care in diabetes-2021. Diabetes Care 2021;44:S211–20. 10.2337/dc21-S015 33298426

[32] von Lewinski D, Kolesnik E, Aziz F et al. Timing of SGLT2i initiation after acute myocardial infarction. Cardiovasc Diabetol 2023;22:269. 10.1186/s12933-023-02000-5 37777743 PMC10544140

[33] Martensson J, Cutuli SL, Osawa EA. Sodium glucose co-transporter-2 inhibitors in intensive care unit patients with type 2 diabetes: a pilot case control study. Crit Care 2023;27:189. 10.1186/s13054-023-04481-y 37194077 PMC10186281

[34] Packer M, Anker SD, Butler J et al. Cardiovascular and renal outcomes with empagliflozin in heart failure. N Engl J Med 2020;383:1413–24. 10.1056/NEJMoa2022190 32865377

[35] Williamson PR, Altman DG, Bagley H et al. The COMET Handbook: version 1.0. Trials 2017;18:280. 10.1186/s13063-017-1978-4 28681707 PMC5499094

[36] Chan AW, Tetzlaff JM, Altman DG et al. SPIRIT 2013 statement: defining standard protocol items for clinical trials. Ann Intern Med 2013;158:200–7. 10.7326/0003-4819-158-3-201302050-00583 23295957 PMC5114123

[37] Boorsma EM, Beusekamp JC, Ter Maaten JM et al. Effects of empagliflozin on renal sodium and glucose handling in patients with acute heart failure. Eur J Heart Fail 2021;23:68–78. 10.1002/ejhf.2066 33251643 PMC8048437

[38] Zarbock A, Kellum JA, Van Aken H et al. Long-term effects of remote ischemic preconditioning on kidney function in high-risk cardiac surgery patients: follow-up results from the RenalRIP trial. Anesthesiology 2017;126:787–98. 10.1097/ALN.0000000000001598 28288051

[39] Chen Y, Wang G, Zhou H et al. 90 days impacts of remote ischemic preconditioning on patients undergoing open total aortic arch replacement: a post-hoc analysis of previous trial. BMC Anesthesiol 2020;20:169. 10.1186/s12871-020-01085-9 32646379 PMC7346644

[40] Hariri G, Collet L, Duarte L et al. Prevention of cardiac surgery-associated acute kidney injury: a systematic review and meta-analysis of non-pharmacological interventions. Crit Care 2023;27:354. 10.1186/s13054-023-04640-1 37700297 PMC10498585

[41] Peng K, Li D, Applegate RL 2nd et al. Effect of dexmedetomidine on cardiac surgery-associated acute kidney injury: a meta-analysis with trial sequential analysis of randomized controlled trials. J Cardiothorac Vasc Anesth 2020;34:603–13. 10.1053/j.jvca.2019.09.011 31587928

[42] Meybohm P, Bein B, Brosteanu O et al. A multicenter trial of remote ischemic preconditioning for heart surgery. N Engl J Med 2015;373:1397–407. 10.1056/NEJMoa1413579 26436208

[43] Tasanarong A, Duangchana S, Sumransurp S et al. Prophylaxis with erythropoietin versus placebo reduces acute kidney injury and neutrophil gelatinase-associated lipocalin in patients undergoing cardiac surgery: a randomized, double-blind controlled trial. BMC Nephrol 2013;14:136. 10.1186/1471-2369-14-136 23829828 PMC3704968

[44] Baysal A, Yanartas M, Dogukan M et al. Levosimendan improves renal outcome in cardiac surgery: a randomized trial. J Cardiothorac Vasc Anesth 2014;28:586–94. 10.1053/j.jvca.2013.09.004 24447501

[45] Zarbock A, Küllmar M, Ostermann M et al. Prevention of cardiac surgery-associated acute kidney injury by implementing the KDIGO guidelines in high-risk patients identified by biomarkers: the PrevAKI-multicenter randomized controlled trial. Anesth Analg 2021;133:292–302. 10.1213/ANE.0000000000005458 33684086

[46] Schurle A, Koyner JL. CSA-AKI: incidence, epidemiology, clinical outcomes, and economic impact. J Clin Med 2021;10:5746. 10.3390/jcm10245746 34945041 PMC8706363

[47] Billings FT 4th, Shaw AD. Clinical trial endpoints in acute kidney injury. Nephron Clin Pract 2014;127:89–93. 10.1159/000363725 25343828 PMC4480222

[48] Kidney Disease: Improving Global Outcomes (KDIGO) Acute Kidney Injury Work Group . KDIGO clinical practice guideline for acute kidney injury. Kidney Int Suppl 2012;2:1–138. 10.1038/kisup.2012.6

[49] Kohler S, Zeller C, Iliev H et al. Safety and tolerability of empagliflozin in patients with type 2 diabetes: pooled analysis of phase I–III clinical trials. Adv Ther 2017;34:1707–26. 10.1007/s12325-017-0573-0 28631216 PMC5504200

[50] Schorling OK, Clark D, Zwiener I et al. Pooled safety and tolerability analysis of empagliflozin in patients with type 2 diabetes mellitus. Adv Ther 2020;37:3463–84. 10.1007/s12325-020-01329-7 32372290 PMC7370973

